# Foot care practice and associated factors among patients with lymphoedema in Boreda district, Gamo zone, southern Ethiopia, 2020. Implications for elimination of podoconiosis and lymphatic filariasis

**DOI:** 10.1186/s13047-021-00490-8

**Published:** 2021-08-10

**Authors:** Chuchu Churko, Tsegaye Yohanes, Alemayehu Bekele Kassahun, Nathan Desalegn, Gesila Endashaw, Mekuria Asnakew Asfaw

**Affiliations:** 1 Collaborative research and training centre for neglected tropical diseases, College of Medicine and Health Sciences, Arba Minch University, Arba Minch, Ethiopia; 2 School of Nursing, College of Medicine and Health Sciences, Arba Minch University, Arba Minch, Ethiopia

**Keywords:** Podoconiosis, Lymphatic filariasis, Foot care practice, Lymphedema, Ethiopia

## Abstract

**Background:**

Lymphatic filariasis is ranked as the second leading cause of disability world-wide. The current global programme to eliminate lymphatic filariasis is based on the interruption of transmission and the alleviation of disability and suffering.

**Objective:**

to assess foot care practice and associated factors among lymphoedema patients in Boreda district, Gamo zone Southern Ethiopia.

**Methods:**

a community based cross sectional study was employed from December 2020 to June 2021 in Boreda district. Simple random sampling technique was used for selecting participants. Pretested structured interviewer administered questionnaire was prepared in English and translated to local language.

**Findings:**

a total of 280 lymphedema patients were involved in this study. More than half 153 (54.6%) had poor practice towards foot care practice with 95% CI (48.7, 60.4%). Patients who fetched 50 l of water or below and wore shoes at the age above 20 years were negatively associated with foot care practice, (AOR = 0.383, 95%CI: 0.155, 0.945) and (AOR = 0.261, 95%CI: 0.107, 0.63), respectively. Patients who owned only one pair and two pairs were negatively associated with foot care practice (AOR = 0.04, 95%CI: 0.009, 0.182) and (AOR = 0.27, 95%CI: 0.087, 0.85), respectively. On the other hand, attending LMMDP service and frequency of adenolymphangitis once and twice or more per month were positively associated with foot care practice (AOR = 3.339, 95%CI: 1.53, 7.285) and (AOR = 8.15, 95% CI: 3.157, 21.058) and (AOR = 9.35, 95% CI: 3.118, 28.059), respectively.

**Conclusion:**

this study indicated foot care practice among lymphedema patients in Boreda district was poor. Number of litre of water collected per day, age at which footwear first worn, number of shoes owned, attending LMMDP and frequency of adenolymphangitis were significantly associated with foot care practice. Standard foot care practice should be emphasized to control progression of lymphedema. Foot care practices like skin care, exercise and elevation, washing legs, bandaging and massaging are important factors that influence in reduction of lymphedema volume and acute attacks among people who are suffering from the diseases.

## Introduction

Podoconiosis and lymphatic filariasis (LF) are neglected tropical diseases (NTDs) that affectthe world’s poorest people and causes a significant economic, social and health burden to developing countries [1]. Podoconiosis or ‘endemic non-filarial elephantiasis’ is a tropical disease caused by exposure of bare feet to irritant alkaline clay soils. This causes an asymmetrical swelling of the feet and lower limbs due to lymphedema. Podoconiosis has a curable pre-elephantiasic phase. However, once elephantiasis is established, podoconiosis persists and may cause lifelong disability. The disease is associated with living in low-income countries in the tropics in regions with high altitude and high seasonal rainfall [2]. On the other hand, lymphatic filariasis or filarial elephantiasis, unlike podoconiosis is transmitted through the bite of an infected mosquito and mostly caused by an agent called *Wuchereria bancrofti* in Africa. It affected more than 36 million people globally and responsible for extremely complex physical, social and economic loss [3].

Lymphatic filariasis also known as filarial elephantiasis has its own impact on health, social and economic well-being of affected population in the world [4]. It is ranked as the second leading permanent disability. Physical and social lives as well as psychological wellbeing of patients with elephantiasis are significantly compromised due to the pain and discomfort, social stigmatization, restricted mobility, feelings of embarrassment and emotional distress that supplement these chronic disfiguring signs [5].

Globally, it is predicted that, 120–129 million people are infected with LF and of these; around 40 million have overt disease, accounting for 5.9 million disability adjusted life years (DALYs), with a concomitant loss of productivity and social stigmatization. Therefore, World Health Organization (WHO) identified LF as a major public health problem and is targeted by for elimination by 2020 [6].

Different literatures throughout the world revealed that practicing foot care has significantly reduced the frequency of acute attack secondary to lymphedema and progression of elephantiasis. Along with secondary infection and the resultant inflammation also seem to play major role in the skin changes seen in the limbs affected by lymphoedema, including the development of elephantiasis [7]. Also, Olszewski reported that simple hygiene, supplemented with antibiotic treatment have profound effect in preventing these acute episodes [8]. Shenoy et al. also demonstrated how well designed programme of foot care such as morbidity management and disability prevention (MMDP) program can significantly decrease the frequency of Adenolymphangitis attacks and also promote to alleviate the disability [9].

In such programmes, meticulous hygiene in treating the affected area needs to be incorporated with the creation of hope and understanding among the patients, their care providers and the community as a whole [10]. As the minimum package of care, managing the lymphoedema seen in LF and podoconiosis patients to manage morbidity is very similar. It is practical that lymphedema morbidity management and disability prevention (LMMDP) should be integrated to help improve cost-effectiveness and extend the reach of the programme [3].

This is particularly important in Ethiopia where there is a high burden of both diseases, with 29 of the 70 LF-endemic districts considered to be co-endemic [11, 12]. Recently training on Lymphedema Morbidity Management and Disability Prevention for health care providers and NTD focals from 20 selected districts of Southern Nation Nationality of People Region (SNNPR), Ethiopia was provided by Arba Minch University collaborative research and training centre for NTDs in collaboration with Ministry of Ethiopia. This will have impact on the way for elimination on podoconiosis by 2030 (i.e. reducing prevalence of podoconiosis below 1%; ensure 100% access to lymphedema management in all endemic districts and Ensure 70% regular shoe wearing and proper foot hygiene practice in all endemic districts) [12, 13].

Despite many efforts done by Ministry of health Ethiopia, burden of podoconiosis still exist in the country with average prevalence of 4% and the highest proportion of cases are found in Southern nation and nationality region accounting prevalence of podoconiosis 8.3%. A more recent mapping data which is conducted in 2018 reported that a total of 16,929 leg lymphoedema was identified in the selected districts of SNNPR; out of this 1084 lymphoedema cases reported in Boreda district, Gamo zone [14].

The high number of leg lymphoedema cases in the current study area highlights the pressing need to deliver a basic package of care to those suffering from these disabling conditions, especially in areas with a high prevalence and/or high density of conditions where patients might be more readily found and the distribution of care easier. In this direction, it was essential to assess the current practices of foot care existing in the patients at community level, and no such information is available in Ethiopia. Hence, the present study was aimed to assess the foot care practices and associated factors among people living with lymphoedema in the rural communities of Gamo zone, SNNPR, Ethiopia, 2020.

## Methods and materials

### Study setting and period

This study was conducted in Boreda district, Gamo zone, Southern Ethiopia from December 2020 to June 2021. Boreda is one of the districts in Gamo zone with high number of elephantiasis case. It is bordered on the Southeast by Mirab Abaya, on the Southwest by Chencha, on the west by Kucha, and on the North by the Wolayita Zone. There is about 1084 lymphoedema cases reported in the study area [14]. The district has 29 kebeles (an aggregate of villages and the smallest administrative unit in Ethiopia) and 5 health centres. Of the total kebeles, 10 were endemic for podoconiosis.

### Study design and eligibility criteria

A community based cross-sectional study design was employed. Individuals who had leg lymphoedema in the study area were eligible for this study whereas severely ill patients were excluded from this study.

### Population

All individuals who have leg lymphoedema in Boreda district were the source population and lymphedema cases in randomly selected kebeles were the study population.

### Sample size and sampling procedure

Sample size was determined using standard procedures by considering the following assumptions: Z_α/2_ = significance level at 95% confidence interval = 1.96; *P* = 50%, to get the highest sample size on practice of foot care among patients; Degree of margin = 5%; 10% non-response rate, the final sample size was 422.

Since total number of cases in the study area was 1084 which is less than 10,000, we used finite population correction formula as follows:

$$\mathrm{Nt}=\mathrm{n}0/1+\mathrm{n}0/\mathrm{N}=422/1+1084=422/1+0.3893=422/1.3893=304=304$$

Hence, the final estimated sample size was 304.

**Fig. 1:**
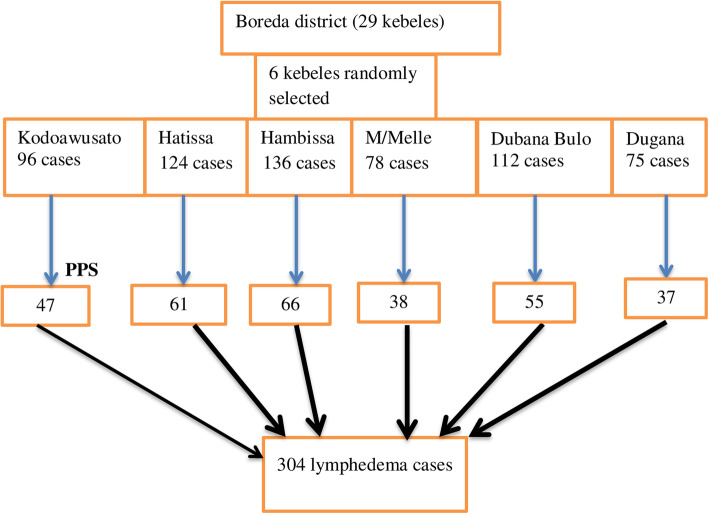
schematic presentation of sampling procedure to assess foot care practice and associated factors among people living with lymphoedema in Boreda district, Gamo zone, southern Ethiopia, 2020

Of the total 10 podoconiosis endemic kebeles, six were randomly selected for data collection. Then number of lymphedema cases in each kebele was identified by health extension workers. The sample size was allocated proportionally to the size of cases in the selected districts. Finally, the study subjects were selected by using systematic sampling technique and selected cases were interviewed based on the pretested structured questionnaire (Fig. 1).

### Variables

#### Dependent variable

- Foot care practice (Good or Poor)

#### Independent variables

- Socio-demographic characteristics, economic factors, environmental factors, health related factors

### Plan for data collection

Data was collected by using pretested structured interviewer administered questionnaire which was developed by reviewing different literatures. Eight data collectors and four supervisors having diploma and above holders with health background and have experience on any research undertakings were recruited for data collection. Each randomly selected patient was subjected to an interview by using a pretested structured questionnaire and asked how she/he dealt with lymphoedema particularly about the treatment, and regular foot care. Subsequently each patient was asked whether she/he practiced the identified foot care measures. We developed nine most commonly recommended foot care measures by reviewing available literatures [15]. A patient was queried on each measure specifically and probed about different foot care practices in her/his daily life. The details of their socio-demographic, economic, personal hygiene and environmental factors, grade and duration of lymphoedema were recorded.

### Operational definition

Lymphoedema: in this study lymphoedema was defined as lymphoedema of lower leg present for more than 1 year in a resident of podoconiosis or lymphatic filariasis endemic area, for which other causes- e.g. onchocerciasis, leprosy, Milroy syndrome, heart and liver failures have been excluded [16].

### Data quality control

The data collection tool was reviewed by team members and then pretested on 5% of sample size outside actual study area. The tool was prepared in English then translated to local language by experienced translators and then back to English to check consistency. Data collectors and supervisors were trained on the tool and familiarized with it. Missing values and inconsistency of the data was checked by supervisors and investigators throughout data collection process.

### Data processing and analysis

After checking completeness of the collected data, entry of data was done by Epi-info version 3.5.1 software and then exported to Statistical Package for Social Sciences (SPSS) version 25 for cleaning, coding and analysis. For quantifying the level of foot care practice, we used nine most commonly recommended foot care measures and the correct answer was given 1 and 0 score for incorrect response. Patients who practiced below the mean of practice question categorized as poor foot care practice and those patients who responded above the mean was considered as good foot care practice. Binary logistic regression analysis was employed to see association of independent variable with outcome variable. Factors which had *p*-value less than 0.25 during bivariate analysis were candidate for multivariate logistic analysis. Statistical significance was shown by 95% confidence interval and p-value<=0.05. Finally, the findings were presented in frequencies, graphs, tables and text.

## Result

### Socio-demographic characteristic of the study subjects

Of the total expected study subjects, complete data were collected from 280 individuals with response rate of 92.1%. All (100%) of the study subjects were living in rural area of Boreda district. One hundred twenty one (43.2%), of the participants are in the age group above 50 years old. Female participants accounted for 169 (60.4%) of the total participants (Table 1).

**Table 1:** Socio-demographic characteristics of lymphedema patients in Boreda district, Gamo zone, SNNPR, Ethiopia, 2021. (*N* = 280): (*others = Merchant and Day labourer**)**

Variables	Category	Frequency	Percentage (%)
Age (in years)	20 and below	13	4.6
	21–30	32	11.4
	31–40	60	21.5
	41–50	54	19.3
	51 and above	121	43.2
Gender	Male	111	39.6
	Female	169	60.4
Religion	Adventist	13	4.6
	Orthodox	78	27.9
	Protestant	189	67.5
Ethnicity	Amhara	15	5.4
	Gamo	263	93.9
	Wolayta	2	0.7
Educational background	No formal education	180	64.3
	Primary education (grade 1–8)	88	31.4
	Secondary and above	12	4.3
Marital status	Divorced	2	0.7
	Married	218	77.9
	Single	30	10.7
	Widowed	30	10.7
Occupation	Farmer	105	37.5
	Housewife	139	49.6
	Student	23	8.2
	Others*	13	4.7
Wealth index	Lowest	61	21.8
	Second	54	19.3
	Middle	97	34.6
	Fourth	17	6.1
	Highest	51	18.2

### Practice of study participants towards lymphedema morbidity management and disability prevention

More than half 153 (54.6%) of the study subjects had poor practice of managing morbidity of lymphoedema with 95% confidence interval of (48.7, 60.4%) (Table 2).

**Table 2:** Practice of study participants towards lymphedema morbidity management and disability prevention, Boreda district, Southern Ethiopia, 2021

Variables	Category	Frequency	Percentage
Washing legs with water and soap daily	Yes	137	48.9
	No	143	51.1
Habit of drying legs after washing	Yes	23	8.2
	No	257	91.8
Habit of cleaning legs apart from washing	Yes	28	10
	No	252	90
Habit of massaging legs	Yes	201	71.8
	No	79	28.2
Habit of elevating legs	Yes	114	40.7
	No	166	59.3
Exercise the affected legs	Yes	74	26.4
	No	206	73.6
Patients worn shoes during interview	Yes	233	83.2
	No	47	16.8
Cleanliness of legs observed	Yes	138	49.3
	No	142	50.7
Patients never walk barefooted	Yes	192	68.6
	No	88	31.4
Overall practice on foot care	Good practice	127	45.4
	Poor practice	153	54.6

### Factors associated with foot care practice among people living with lymphoedema

In bivariate analysis, wealth index, number of litres collected per day, age at which shoes worn, number of shoes owned, duration of swelling occupation, age category, attended LMMDP treatment, experienced acute attack and frequency of acute attack became *p*-value less than 0.25 and therefore, were candidate for multivariate logistic regression model.

After adjusting for other variables, number of litre collected per day per house, age at which shoes first worn, number of shoes owned, attended LMMDP treatment and frequency of acute attack were significantly associated with foot care practice. Patients who collected 50 l or below water were 38% less likely to have good foot care practice when compared to those patients who collected more than 50 l of water per day (AOR = 0.383, 95%CI: 0.155, 0.945). Study subjects who wore shoes at the age above 20 years were 26% less likely of having good foot care practice as compared to patients who wore shoes at age 20 years or below (AOR = 0.261, 95%CI: 0.107, 0.63). On the other hand, patients who owned only one pair and two pairs were 4 and 27% less likely to have good foot care practice in comparison with three or more pairs of shoes owned (AOR = 0.04, 95%CI: 0.009, 0.182) and (AOR = 0.27, 95%CI: 0.087, 0.85), respectively.

According to this study, participants who attended LMMDP service were 3 times more chance to have good foot care practice than those who did not attend the service (AOR = 3.339, 95%CI: 1.53, 7.285). Likewise, frequent acute attack of adenolymphangitis was statistically significantly associated with good practice of foot care (AOR = 8.15, 95% CI: 3.157, 21.058) and (AOR = 9.35, 95% CI: 3.118, 28.059) (Table 3).

**Table 3:** Factors associated with foot care practice among lymphoedema patients in Boreda district, Gamo zone, Southern Ethiopia, 2021. (*significantly associated with foot care practice; **strongly associated with foot care practice**)**

Variables	Category	Status of foot care practice		COR with 95%CI	AOR with 95%CI	*P*-values
		Good	Poor			
Wealth index	First percentile	31	30	3.029 (1.35, 6.758)	2.621 (0.838, 8.193)	0.098
	Second percentile	32	22	4.25 (1.85, 9.76)	1.213 (0.376, 3.908)	0.747
	Middle percentile	45	52	2.53 (1.2, 5.3)	1.468 (0.533, 4.042)	0.457
	Fourth percentile	6	11	1.59 (0.49, 5.175)	0.261 (0.047, 1.431)	0.122
	Fifth percentile	13	38	-		
Number of litres of water fetched per day	50 or below	79	133	0.247 (0.137, 0.447)	0.383 (0.155, 0.945)	0.037*
	Above 50 l	48	20	Reference	-	
Age at which shoes first worn	20 or years old	108	88	Reference		
	Above 20 years old	19	65	0.238 (0.133, 0.427)	0.261 (0.107, 0.63)	0.003*
Number of shoes owned	Only one pair of shoes	15	40	0.167 (0.068, 0.41)	0.04 (0.009, 0.182)	0.001**
	Two pairs of shoes	85	101	0.374 (0.179, 0.783)	0.27 (0.087, 0.85)	0.026*
	Three or more pairs	27	12	Reference	-	
Attended LMMDP treatment	No	30	86	Reference		
	Yes	97	67	4.15 (2.469, 6.976)	3.339 (1.53, 7.285)	0.002*
Frequency of acute attack	Every month	50	18	6.77 (3.514, 13.028)	8.15 (3.157, 21.058)	0.001**
	Twice or more in a month	29	12	5.89 (2.73, 12.7)	9.35 (3.118, 28.059)	0.001**
	Once per year	39	95	Reference		

### Home-environmental characteristics of lymphedema patients

Eighty nine (31.8%) of the study subjects said that one round walking distance to fetch safe drinking water was more than 30 min and more than three fourths fetch less than 50 l of water per day. Majority, 264 (94.3%) of the patients had functional pit latrine (Table 4).

**Table 4:** Home-environmental characteristics of the study participants, *N* = 280

Variables	Category	Frequency	Percentage (%)
Distance from home to safe drinking water (one round walking distance in minute)	30 min and below	191	68.2
	Above 30 min	89	31.8
Number of litres of drinking water fetched per day	50 or below	212	75.7
	Above 50	68	24.3
Latrine conditions	No latrine	3	1.1
	Functional pit latrine	264	94.3
	Non-functional latrine	13	4.6
Weather conditions	Mid-land	44	15.7
	lowland	236	84.3
Type of soil	Red clay soil	228	81.4
	Sandy soil	13	4.6
	Black soil	39	14

### Personal hygiene behaviour of lymphoedema patients in the study area

Regarding personal hygiene, 167 (59.6%) of the study subjects never walk barefooted whereas 33 (11.8%) and 73 (26.1%) become barefooted when farming and at home respectively. Most 196 (70%) of study participants started wearing shoes at the age of 20 years or below (Table 5).

**Table 5:** Personal hygiene behaviour of lymphoedema patients in the study area, *N* = 280

Variables	Category	Frequency	Percentage (%)
Situations in which patients walk barefooted	Never barefooted	167	59.6
	No habit of shoe wearing	7	2.5
	Barefooted when farming	33	11.8
	Barefooted when at home	73	26.1
Age at which shoes worn	20 year or below	196	70
	Above 20 year	84	30
Number of pairs of shoes owned	Only one pair	55	19.6
	Two pair	186	66.4
	Three or more pair	39	14
Number of pairs of shoes needed per year	4 or less pairs	177	63.2
	Above 4 pairs	103	36.8
Conditions where patients take care their legs	Wash legs with water and soap	137	49
	Wash legs with water only	56	20
	No habit of washing legs	0	0
	Washed legs last night	246	87.9
	Washed lags daily last week	215	76.8

### Clinical history and characteristics of patients

Thirteen (4.6%) of the respondents have wounds on their legs. Of those who had wounds on their legs, 9 (69.2%) did not clean the wound (Table 6).

**Table 6:** Clinical history and personal hygiene characteristics of patients, *N* = 280

Variables	Category	Frequency	Percentage
Wounds present on the affected legs	Yes	13	4.6
	No	267	95.4
If wounds present, do you clean the wound (*N* = 13)	Yes	9	69.2
	No	4	30.8
Experienced acute attack of the affected leg	Yes	243	86.8
	No	37	13.2
If experienced ALA, knew cause of acute attack (*N* = 243)	Don’t know	43	17.7
	When walking long distance for hours	48	19.7
	When weather condition changes	152	62.6
Frequency of ALA occurrence, (*N* = 243)	Monthly	68	28
	More than once a month	41	16.9
	Every year	134	55.1
Legs affected	Both legs	243	86.8
	Only one leg	37	13.2
Progression of swelling	Don’t remember	39	13.9
	From down to up knee	226	80.7
	From hip to down	15	5.4
Duration of swelling	20 years or less	170	60.7
	21–40 years	99	35.4
	41 and above years	11	3.9
Family history of leg swelling	Yes	59	21.1
	No	221	78.9
Number of family member affected (*N* = 59)	One	41	69.5
	Two	12	20.3
	Three	2	3.4
	Four	4	6.8
Relationship with affected family member (one patient might have more than one family member affected, therefore total sum of percent became more than 100%)	Father	15	25.4
	Mother	19	32.2
	Child	25	42.4
	Sister	8	13.6
	Brother	7	11.9
	Grand family	8	13.6
Patient worn shoes at the time of interview	Yes	233	83.2
	No	47	16.8
Type of shoes patient worn at the time of interview (*N* = 233)	Canvas	161	69.1
	Fully covered leather	2	0.9
	Hard plastic	1	0.4
	Open shoes	69	29.6
Cleanliness of legs	Clean and intact	138	49.3
	Not clean	142	50.7
Odor of the leg	No odor	213	76.1
	Has bad odor	67	23.9

### Accessibility to health care service

Of the total respondents, 116 (41.4%) did not attend LMMDP service because of different reason like didn’t want 62(53.5%), health facility far from home 7 (6%) and no treatment centre available 47 (40.5%) (Table 7).

**Table 7:** Accessibility of the respondents for health care services in Boreda district, Southern Ethiopia, 2021

Variables	Category	Frequency	Percentage
Patients attend lymphoedema management service	Yes	164	58.6
	No	116	41.4
Reasons for not attending LMMDP service (*N* = 116)	Didn’t want	62	53.5
	Health facility far from home	7	6
	No treatment centre in the area	47	40.5
Type of health facility attended (*N* = 164)	Health centre	154	93.9
	Hospital	10	6.1
Trained on self-care management of foots (*N* = 164)	Yes	94	57.3
	No	70	42.7
Graduated from health extension packages?	Yes	17	6.1
	No	263	93.9

## Discussion

This community based cross sectional study was conducted to assess foot care practice and associated factors among people living with lymphedema in Boreda district, Southern Ethiopia. The result of this study found that more than half (54.6%) of study subjects had poor foot care practice. In contrast, a study done in Wolayta zone in 2016 showed that about 25.9% of the study subjects did not practiced stepwise treatment procedures provided by Mossy Foot Association health workers [17]. This difference might be due to in our study area patients had less awareness and not well understood importance of practicing foot care and no much effort has been done to increase foot care practice.

In this study we found that patients who collected 50 l or below water per day were 38% less likely to have good practice of foot care when compared to those who collected more than 50 l of water per day. The possible reason might be the fact that accessibility to water is closely linked with personal hygiene practices i.e. the more they collect water daily, the more they take care of their foot. Ministry of health Ethiopia also recognized that high number of NTDs burden is linked with inadequate hygiene and sanitation conditions, and it is now recognized globally that prevention and treatment of NTDs must include water, hygiene and sanitation improvements in addition to mass drug administration [18].

According to this study the study subjects who used footwear first at the age of above 20 years old were 26% times less to practice foot care measures when compared to the counterpart. The lower the age of patients to begin footwear, the better patients take care of their foot. This might indicate that people who started shoe wearing at early ages take care of themselves than others. To our knowledge, there is no previous literature conducted to discuss with this finding. This result is the first in its kind.

The present study revealed that number of shoes owned by the patients was significantly associated with foot care practice. Those study subjects who owned only one and two shoes were 4 and 27% less likely to practice foot care in comparison with patients with three or more shoes owned, respectively. This result might indicate the more number of shoes owned by the patients the better they take care of their foot. In addition to that those patients who owned three or more pairs of shoes might be in a good economic status and had access to services of lymphedema and afford consumables easily for their feet.

We found that frequency of acute attack was significantly strongly associated with good practice of foot care. Those patients who experienced one and more than one acute attack per month were 8 and 9 times more likely to have good foot care practice than patients who faced one acute adenolymphangitis per year. The possible reason might be patients with more frequent acute attack of adenolymphangitis visit clinics for seeking treatment of the pain and thereby obtain information regarding foot care.

Although this study is the first in its kind to associate foot care practice with other variables, it has the following limitations: there might be recall bias regarding frequency of acute attack in a year and might not remember the exact age at which shoes first worn. This finding may not be generalizable to lymphoedema patients in Gamo zone because it is limited to Boreda district. In this study we did not use appropriate theory for designing questionnaire and is one of the potential weakness. Hence, acceptable theory for questionnaire design is recommended for future study [19].

## Conclusion and recommendation

In conclusion, this study indicated foot care practice among people living with lymphoedema in Boreda district is poor. More than half of the study subjects had poor practice regarding foot care measures. In multivariable regression analysis variables like number of litre of water fetched per day, age at which used footwear first, number of shoes owned, attending LMMDP treatment and frequency of acute attack were significantly associated with foot care practice. Therefore, standard foot care practice should be emphasized to control progression of lymphedema and improve care of patients towards practice measures. Shoe wearing habit at early age and other preventive strategies on podoconiosis and lymphatic filariasis should be practiced and implemented at Boreda district.

## Data Availability

The datasets during and/or analysed during the current study are available from the corresponding author on reasonable request.

## Abbreviations

AOR: Adjusted Odds Ratio
CI: Confidence Interval
CMHS: College of Medicine and Health Sciences
DALYs: Disability Adjusted Life Years
LF: Lymphatic Filariasis
LMMDP: Lymphedema Morbidity Management and Disability Prevention
NTDs: Neglected Tropical Diseases
PPS: Proportional to Size
SNNPR: Southern Nation Nationality People Region
SPSS: Statistical Package for Social Sciences
WHO: World Health Organization
